# Nurturing a lexical legacy: reading experience is critical for the development of word reading skill

**DOI:** 10.1038/s41539-017-0004-7

**Published:** 2017-01-27

**Authors:** Kate Nation

**Affiliations:** University of Oxford, Oxford, UK

## Abstract

The scientific study of reading has taught us much about the beginnings of reading in childhood, with clear evidence that the gateway to reading opens when children are able to decode, or ‘sound out’ written words. Similarly, there is a large evidence base charting the cognitive processes that characterise skilled word recognition in adults. Less understood is how children develop word reading expertise. Once basic reading skills are in place, what factors are critical for children to move from novice to expert? This paper outlines the role of reading experience in this transition. Encountering individual words in text provides opportunities for children to refine their knowledge about how spelling represents spoken language. Alongside this, however, reading experience provides much more than repeated exposure to individual words in isolation. According to the lexical legacy perspective, outlined in this paper, experiencing words in diverse and meaningful language environments is critical for the development of word reading skill. At its heart is the idea that reading provides exposure to words in many different contexts, episodes and experiences which, over time, sum to a rich and nuanced database about their lexical history within an individual’s experience. These rich and diverse encounters bring about local variation at the word level: a lexical legacy that is measurable during word reading behaviour, even in skilled adults.

Most children cannot read before they go to school, but fast forward a few years and they are working their way through Harry Potter. How does this learning happen? The science of reading has taught us much about the genesis of reading. In alphabetic languages such as English, we know that an understanding of phonology—the sound system of spoken language—underpins the development of the alphabetic principle^1^—the insight that print represents meaning via sound. Armed with this insight, children discover the spelling-sound mappings that characterise their language and from this, they have a means to access language from print. Our scientific understanding of the end point of learning is also advanced. Cognitive psychology is abound with studies examining how adults process written words^2^ and much is known about the neural systems that support word reading.^3–5^ Despite a rich understanding of both beginning reading and its end state, how children move from one to the other is not well understood. The lexical legacy hypothesis, introduced in this paper, provides a new perspective on the transition from novice to expert.

The focus of this paper is with how people read words. While reading comprehension requires much more than the identification of individual words, comprehension can not happen without it.^6,7^ Thus, understanding how word reading expertise develops is critical. Importantly, however, as will become clear once the lexical legacy hypothesis is described, we can not divorce the processes involved in word identification from the reality that words are not experienced in an isolated vacuum. Words occur in meaningful context, in both spoken and written language. The lexical legacy account argues that this is important. It sees skilled word reading as, in part, a consequence of experiencing words in diverse and meaningful language environments during reading experience. Reading experience provides the substrate that allows a person to build knowledge of an individual word, not just of its spelling and pronunciation, but knowledge of its meaning and how it connects to other words. This rich knowledge base underpins reading fluency and reading comprehension. But before elaborating further, we need to begin with what happens before then, for reading experience can only exert its influence once children are able to read words. So what needs to happen to get the system kick-started?

## Beginning reading: from overt phonological decoding to orthographic processing

Compare your experience of reading words with that of a young child. For you, word reading is usually fast, accurate and largely without effort. For a novice, reading is characterised by phonological decoding, whereby letter strings are closely analysed and laboriously ‘sounded out’ to form words. This moment of introspection highlights the essence of what needs to develop: word reading becomes more automatic and less effortful. How is this achieved?

Let us first focus on beginning reading. There is clear consensus and abundant evidence (for review, see refs 1,8,9) that in alphabetic languages, phonological decoding is at the core of learning to read words. Put simply, learning how letters (or graphemes) relate to sounds (or phonemes) allows children to begin to learn the skills required to access the spoken form of a word from its written form. This takes time to develop and requires instruction and practice. Initially, decoding attempts may be only partially correct and certainly will be effortful; with practice in applying their knowledge of grapheme-phoneme relations to read words, children’s decoding skills improve and reading becomes more fluent. According to Ehri’s phase theory of reading development, learning to decode is a connection-forming process in which the spelling patterns of words become tightly bonded with their pronunciations. These unitised representations are retained in memory, supporting efficient visual word recognition and access to meaning.^8^

Share’s self-teaching hypothesis also has phonological decoding at the foundation of learning to read—indeed, he describes it as indispensible and absolutely necessary: the *sine qua non* of reading acquisition.^9^ Why is this the case? According to Share, although phonological decoding might initially be effortful and laborious, by forcing the translation from print to sound, it provides an opportunity to acquire word-specific orthographic information about the word, its spelling pattern and its pronunciation. This will then be available on future encounters with the word, lessening the reliance on overt and effortful phonological decoding. As well as word-specific knowledge, this process supplies children with a means to gradually accumulate knowledge about how their orthography (that is, their writing system) works. This might include knowledge of regularities and sub-regularities, orthographic conventions and exceptions to those conventions, statistics, which sum over time to provide each child with their own experience-based database of orthographic knowledge. A good deal of evidence supports the central aspects of the self-teaching hypothesis, including its critical foundation in phonological decoding and how it facilitates word reading development;^10–12^ it is also supported by a computational implementation.^13^

The self-teaching hypothesis describes how a small system can expand rapidly. It is, however, largely silent as to how orthographic expertise develops. The basics of word reading in hand, the task ahead is nevertheless enormous: with just 26 letters to represent the many thousands of written words we encounter, the amount of orthographic overlap between words is considerable.^14^ Experiments with adults show how words interact and compete with each other during processing, pointing to a system that is tuned to be highly efficient at getting us from print to meaning quickly.^2^ In terms of development, as overt and serial decoding declines, more automatic and parallel phonological activation from print emerges and indeed, this remains a stable feature of skilled word recognition.^15–17^ Alongside this, development brings critical changes in orthographic processing, with evidence that coarse-grained orthographic coding increases with reading level, as the system becomes more adult like.^15^ It is likely that children also develop increased sensitivity to morphological complexities and regularities, allowing them to capitalise on the relationships between a word’s morphological structure and its spelling.^18–20^ Clearly, something develops as children move from novice to expert, and while this has its roots in phonological decoding, much more research on the development of orthographic expertise is needed.^10^

## Reading experience and the development of lexical quality

Perfetti’s lexical quality hypothesis^6,21^ defines lexical quality as the extent to which a word's mental representation specifies its spelling, sound and meaning. High quality representations contain tightly bound orthographic, phonological and semantic constituents that together comprise a word’s identity. Higher quality representations are considered to be more fully-specified, more stable and less context-bound than those of lower quality. As a result, they support efficient word identification during reading, freeing cognitive resources for the ultimate purpose of reading: comprehension.

The lexical quality hypothesis argues that for all of us, there are words we know well and others we know less well. Greater expertise is associated with a higher mean lexical quality: on average, adults will have a higher mean lexical quality than young children. An attractive feature of the lexical quality hypothesis is that it unites ideas about knowledge with ideas about cognitive processing. Knowledge is represented by lexical quality and differences in lexical quality lead to differences in processing. In turn, effective processing provides opportunities to gain knowledge and this serves to further tune lexical quality to the benefit of future processing. In this way, lexical quality is both a cause and a consequence of individual and developmental differences in reading skill, although the mechanisms that bring about change in lexical quality are not yet detailed.^6^

This brings us to what has to be the broad answer to the novice-expert question: experience. Reading is a skill and like other skills, practice is critical to gaining expertise. Once phonological decoding is in place, practice allows basic skills to be honed and reading experience provides the substrate from which lexical processes can be tuned to the specific orthography being learned. This fits with the finding that print exposure (estimates of how much an individual reads) is a powerful predictor not just of reading outcomes in children,^22^ but of word reading processes in skilled adults too.^23,24^

## The lexical legacy hypothesis

Reading experience provides opportunities to refine knowledge about orthography-phonology mappings. Importantly though, it provides much more than repeated exposure to individual words: words are usually encountered in meaningful sentences, stories and texts. What is the relevance of this type of experience? The lexical legacy hypothesis sees it as critical to variations in lexical quality. At its heart is the idea that reading (and spoken language) provides many different contexts, episodes and experiences which, over time, sum to a rich and nuanced database about a word, its connections to other words and its lexical history within an individual’s experience. The hypothesis suggests that these rich and diverse encounters bring about local variation at the word level: a lexical legacy that is measurable during word reading behaviour.

This account is related in spirit to theories of word knowledge based on lexical co-occurrence and the principle that “you shall know a word by the company it keeps.”^25^ Mathematical models of word knowledge based on latent semantic analysis demonstrate the utility and psychological validity of this statistical approach to meaning, in which words come to occupy a position in semantic space, based on encounters during the course of language experience. Put simply, a word’s position in semantic space at any one point in time, relative to other words, captures its meaning (for reviews see refs 26,27). The extension of this approach to the development of word *reading* (rather than meaning) is speculative, but several lines of evidence converge to suggest it is one worth exploring. To illustrate its utility, consider the frequency effect.

Frequency (how often a word appears in a language corpus) enjoys special status as a powerful item-level predictor of lexical processing.^28,29^ It is represented in models of word recognition in various ways, consistent with the notion that seeing a word more frequently adjusts its recognition threshold so that it is more easily processed on subsequent encounters. Clearly, frequency is the product of cumulative experience. Some words are seen more often than others. Less clear, however, is whether the frequency effect arises solely from variations in repetition. How often a word appears in a corpus is correlated with many other factors, including the local semantic and syntactic contexts in which the word appears throughout the corpus. This type of linguistic co-occurrence predicts the frequency effect,^30^ suggesting that frequency might serve as an umbrella for complex lexical experience. On this view, frequency is a powerful predictor of reading as it subsumes other features, co-captured by experience,^31^ as well as being an indicator of repetition in and of itself.

Consistent with a word’s frequency representing more than just the number of times it is likely to have been seen, the number of unique documents a word appears in is more closely associated with lexical processing in adults than raw frequency.^32^ And, it seems likely that it is the semantic diversity of those different contexts that matters most: words experienced in more varied semantic contexts enjoy a processing advantage in word reading and lexical decision,^31,33–36^ relative to words of equivalent frequency that occur in more redundant contexts (lexical decision is a commonly used psycholinguistic task in which participants respond yes if a stimulus is a word and no if it is a pseudoword). One way to interpret this finding, supported by both behavioural and computational evidence,^33,36^ is that change is needed to bring about learning. Simply repeating a word in isolation or across identical documents will not update a word’s lexical history; in contrast, differences in linguistic environment associated with a changing semantic context will cause the word’s lexical representation to be updated, and so enhance learning.

This interpretation of the frequency effect requires us to rethink what frequency captures and why it is an important item-level predictor of word reading. To return to the lexical legacy hypothesis, semantic diversity might be relevant beyond raw frequency as it captures the linguistic environment a word has been experienced in, with variations in this being reflected in lexical quality. Other item-level predictors might exert their influence for similar reasons. Consider classic semantic variables such as imageability, number of semantic features and number of senses. These variables are also associated with the ease of word recognition in skilled readers.^28,37–39^ This might be the measurable legacy that follows from reading experience, where instances with words in meaningful text brings about differences in lexical quality.

## Questions to ask about the lexical legacy hypothesis

The alphabetic principle underpins word reading development. Once the basics are in place, further development comes from reading experience. The lexical legacy hypothesis helps us to understand how this input allows distributional information to be massed over time, influencing reading skill. A number of features make this hypothesis attractive. It offers a means by which differences in lexical quality emerge. It helps us to understand the relationship between word reading skill and print exposure. It also forges direct links between lexical learning and lexical processing: how easily a word is processed, even by skilled adults, is a product of the learning opportunities afforded by an individual’s lexical experience. It is, however, speculative and under-specified. I end by setting out some questions that need to be addressed.

Most current data investigating the relationship between linguistic experience and lexical processing are limited by their correlational nature, as in the correlation between print exposure and reading development noted earlier, for example refs 22–24. To move beyond this, studies that explicitly manipulate and control variables are needed. Encouragingly, training experiments with adults suggest that linguistic diversity supports learning.^33,36,40^ Extending this work to children will shed light on whether diversity has relevance for how children learn written words. If the lexical legacy hypothesis is correct, experiencing words in more diverse linguistic contexts should bring about better learning of orthographic forms. Training studies that control for frequency of exposure to each new word while manipulating the number or nature of contexts or episodes each is experienced in will be particularly informative. For example, children could read some novel words embedded in a series of stories where the context varies from story to story. If diversity is more critical than repetition, experiencing words in different contexts should result in better learning than when the novel words are encountered the same number of times, but in non-diverse contexts. A related set of questions is what is meant by diverse contexts: is it variation in the number of contexts (for example, the number of different books a child sees a word in) or the temporal spacing of the encounters (for example, three times on one day vs. once per day over three days). Or, is it more about the nature of the linguistic context that characterises each encounter, and the similarity of those contexts to previous lexical experiences? Again, carefully designed training experiments have utility here, as do computational models.

Clearly, word knowledge is multifaceted. We may wish to know how well a child has learned the written form of the word; or, we may be more interested in how well meaning has been acquired; or, we may be interested in how readily word meaning is activated from written forms when children read words in text. Regardless of our question of focus, sensitive measures of learning will be needed to tap knowledge that is partial and incremental, as it builds over time with each exposure.^10,41,42^ Comparing learning across different exposure conditions should then reveal what type of experience is optimal to bring about learning. Using this type of design, a recent training experiment found that children better learned the meaning of new verbs when they were experienced in episodes built around a common scenario.^43^ Potentially, this type of contextual experience promoted semantic connections between words and in doing so, promoted learning of meaning.

Another question is whether it is reading experience that matters for providing diversity, rather than language experience more generally. While spoken language experience is of course relevant, there are reasons to propose that experience with text also plays a role. Once children are able to read, the majority of new vocabulary is learned via reading, not listening.^44^ Even text written for young children is more lexically diverse than speech,^45^ and there are differences in syntax too,^46^ consistent with the idea that experience with text affords unique learning opportunities. And text is clearly needed for children to learn and refine their knowledge about how spelling patterns relate to spoken language: word reading skill demands efficient mappings between orthography and phonology,^47^ as well as orthography and morphology,^18,19^ tuned to the individual’s language system.^48^ One way to investigate the impact of spoken vs. written language experience on reading development would be to extract lexical statistics such as frequency and semantic diversity from corpora that sample either children’s spoken or written language experience, respectively. If linguistic experience gained via text is important, the item-level association between reading behaviour and text-corpora statistics should be closer than its association with spoken-corpora statistics.

In closing, it is important to emphasise what the hypothesis is not saying. It is not the case that young children learn to read words by contextual guessing, as is clear from a large evidence base.^49–52^ To the contrary, the foundation of learning to read in English is the alphabetic principle and from this, the development of high quality phonological decoding skill.^1,8,9^ This provides the means for orthographic learning—the gradual accumulation of orthographic knowledge, via reading experience.^9–12^ Building on this, the lexical legacy hypothesis situates expertise as the product of reading experience in a broader sense. How often words occur, how they are used, how they look and sound, what they come to mean and how they relate to other words all feed into a dynamic database of knowledge, continuously updated by experience. Reading behaviour, for an individual word averaged over people, or an individual person averaged over words, is the product of this rich experience at that point in time.
